# Association Between Common Infections and Incident Post-Stroke Dementia: A Cohort Study Using the Clinical Practice Research Datalink

**DOI:** 10.2147/CLEP.S260243

**Published:** 2020-08-21

**Authors:** Caroline E Morton, Harriet J Forbes, Neil Pearce, Liam Smeeth, Charlotte Warren-Gash

**Affiliations:** 1 Department of Non-Communicable Disease Epidemiology, London School of Hygiene and Tropical Medicine, London WC1E 7HT, UK; 2 EBM DataLab, Nuffield Department of Primary Care Health Sciences, University of Oxford, Oxford OX2 6GG, UK; 3 Department of Medical Statistics, London School of Hygiene and Tropical Medicine, London WC1E 7HT, UK

**Keywords:** post-stroke dementia, infections, cohort study, electronic health records

## Abstract

**Purpose:**

To investigate the association between common infections and post-stroke dementia in a UK population-based cohort.

**Materials and Methods:**

A total of 60,392 stroke survivors (51.2% male, median age 74.3 years, IQR 63.9–82.4 years) were identified using primary care records from the Clinical Practice Research Datalink (CPRD) linked to Hospital Episode Statistics (HES) with no history of dementia. Primary exposure was any GP-recorded infection (lower respiratory tract infection (LRTI), urinary tract infection (UTI) requiring antibiotics, skin and soft tissue infection requiring antibiotics) occurring after stroke. The primary outcome was incident all-cause dementia recorded in primary care records. In sensitivity analyses, we restricted to individuals with linked hospital records and expanded definitions to include ICD-10 coded hospital admissions. We used multivariable Cox regression to investigate the association between common infections and dementia occurring from 3 months to 5 years after stroke.

**Results:**

Of 60,392 stroke survivors, 20,969 (34.7%) experienced at least one infection and overall 4512 (7.5%) developed dementia during follow-up. Early dementia (3 months to 1-year post-stroke) risk was increased in those with at least one GP-recorded infection (HR 1.44, 95% CI 1.21–1.71), with stronger associations when hospitalised infections were included (HR 1.84, 95% CI 1.58–2.14). Late dementia (1–5 years) was only associated with hospitalised, but not with GP-recorded, infections.

**Conclusion:**

There was evidence of an association between common infections and post-stroke dementia, strongest in the 3–12 months following stroke. Better understanding of this relationship could help inform knowledge of pathways to dementia post-stroke and targeting of preventive interventions.

## Introduction

There are over 1.2 million stroke survivors in the UK,^1^ where stroke is the commonest cause of complex disability. Cognitive problems contribute substantially to post-stroke disability, and while physical impairments will often improve after stroke, cognitive function may worsen with time. The incidence of post-stroke dementia typically ranges from 7–20% in community-based studies, but may reach 40% in hospital-based studies.^2^ Comparisons between studies are limited by differing definitions and study populations.

Risk factors for post-stroke dementia include demographic factors (older age, female sex, non-White ethnicity), existing cognitive decline, location and type of stroke, as well as the presence of small vessel disease or cortical atrophy on neuroimaging.^2^–^4^ Delirium and early seizures^5^ after stroke are associated with dementia risk,^5^,^6^ although these factors suggest increased stroke severity, itself a risk factor for post-stroke dementia. The role of common infections in dementia risk is debated. Infections could act through systemic inflammatory pathways to trigger a disordered microglial response in the aged brain.^7^ While infections in stroke survivors may indicate a more severe stroke, they also occur after mild strokes. It is unclear whether type, frequency or severity of infections affects dementia risk.

Better understanding of the relationship between infections and dementia in the post-stroke setting will help to inform development and targeting of interventions to preserve cognitive function after stroke. We therefore aimed to investigate the association between common infections and post-stroke dementia in a UK population cohort using linked health data.

## Materials and Methods

### Data Sources and Study Design

We conducted a cohort study using the Clinical Practice Research Datalink (CPRD). CPRD Gold contains anonymised GP records of Read-coded diagnoses, investigations, prescriptions and referrals, patient demographics and registration information. Covering around 8% of the UK population, it is broadly representative by age, gender, ethnicity and mortality.^8^ Around 75% of English CPRD practices are linked to Hospital Episode Statistics (HES). The HES Admitted Patient Care database contains ICD-10 coded records of admissions to all NHS hospitals in England from 1997.^9^

### Study Population

We included adults aged ≥40 years who survived an incident first stroke occurring from 01/01/2005-31/12/2016, with at least 1 year of follow-up prior to stroke. Stroke was defined as the first record of any stroke type (ischaemic, haemorrhagic or unspecified) in CPRD or HES according to codelists developed and refined with clinicians. Stroke-specific diagnostic codes in EHRs have a high validity (positive predictive value≥90%).^10^ We excluded individuals with dementia before stroke and those who died or had a new dementia record in the 3 months after stroke.

### Definition of Exposure and Outcome

The primary exposure was GP-recorded infection occurring after stroke, defined as lower respiratory tract infection (LRTI), urinary tract infection (UTI) treated with antibiotics or skin/soft tissue infection (SSTI) treated with antibiotics. An algorithm for identifying infections that built upon existing codelists^11^–^13^ was developed and reviewed by two practising clinicians (appendix 1). Prescription data was used to identify antibiotics given on the day of infection. Exposure status was time-varying, so patients contributed person time to the unexposed category until exposure to an infection, at which point they contributed person time to the exposed category. In sensitivity analyses of patients with linked hospital data, the exposure definition was expanded to include ICD-10 coded infections occurring in hospital (an infection code in any position in the record) as well as community-acquired infections resulting in hospitalisation (an infection recorded as the primary diagnosis). In secondary analyses, we explored the effects of number and type of infections, with multiple codes occurring within 28 days considered to relate to the same infectious episode.

The primary outcome was all-cause dementia, identified through clinical Read codes in CPRD (or ICD-10 codes in HES for analyses restricted to those with linked data) dated from the first record (appendix 2). Dementia was divided into early post-stroke dementia (3 months to 1 year) and late post-stroke dementia (1 to 5 years). We focused on dementia occurring from 3 months after stroke to avoid misclassifying delirium or other reversible cognitive changes associated with stroke as dementia.^2^ Patients whose first dementia code suggested prevalent (rather than incident) dementia were excluded.

### Definition of Covariates

We extracted data on age and sex, and identified categories of ethnicity from CPRD and HES data using an approach previously described.^14^,^15^ Socio-economic status was assessed at practice level using quintiles of the Index of Multiple Deprivation 2016.^16^ History of depression, diabetes, myocardial infarction and atrial fibrillation before stroke was defined using CPRD clinical diagnostic codes. Use of statins, blood-pressure lowering medication, and immunosuppressant medication in the two years prior to the stroke was identified from CPRD prescription records. Baseline alcohol use, smoking status and the nearest BMI measurement prior to stroke were also taken from CPRD. Prescription of an anti-platelet agent within 90 days of stroke was identified from CPRD prescription records. We identified new depression diagnoses by depression Read codes following stroke, in patients not coded with depression in the two years prior to stroke. Second or subsequent strokes were identified using ICD-10 codes in the primary diagnosis field in HES, with repeated stroke codes within 28 days considered part of the same episode. We did not consider repeated CPRD stroke codes as second episodes, as multiple GP consultations in the post-stroke period may include stroke codes but are unlikely to represent acute events.

### Statistical Analysis

Follow-up began at 3 months after stroke and ended at the earliest of incident dementia, death, transfer out, practice final data collection date, 5 years post-stroke or 31/12/2016. We described the study population at time of stroke and compared characteristics by exposure and outcome variables. We calculated rates of early and late dementia per 1,000 person years at risk (PYAR) with 95% confidence intervals for each exposure category. We developed multivariable Cox regression models to assess the relationship between the time-updated primary exposure any GP-recorded infection and early and late dementia using CPRD data only, first adjusting for age and sex, and then including additional covariates. We carried out several sensitivity analyses restricted to patients with linked CPRD-HES data, which included: 1) repeating the primary analysis in patients with linked data to increase ascertainment of dementia; 2) expanding the definition of infection to include infections recorded in either GP or hospital records (any position), or in GP or hospital records (first position only); and 3) repeating all analyses excluding infections occurring within 3 months of stroke. We then explored the effect of number of infection episodes and first infection type. Likelihood ratio tests were used to calculate p-values. Analyses were carried out using Stata version 15. Analytic code is available at GitHub (https://github.com/CarolineMorton/stroke-infection-research-code).

### Ethics Statement

This study was approved by CPRD’s Independent Scientific Advisory Committee (17_176R) and the London School of Hygiene & Tropical Medicine Research Ethics Committee (14,319).

## Results

### Description of Study Cohort

**Figure 1:**
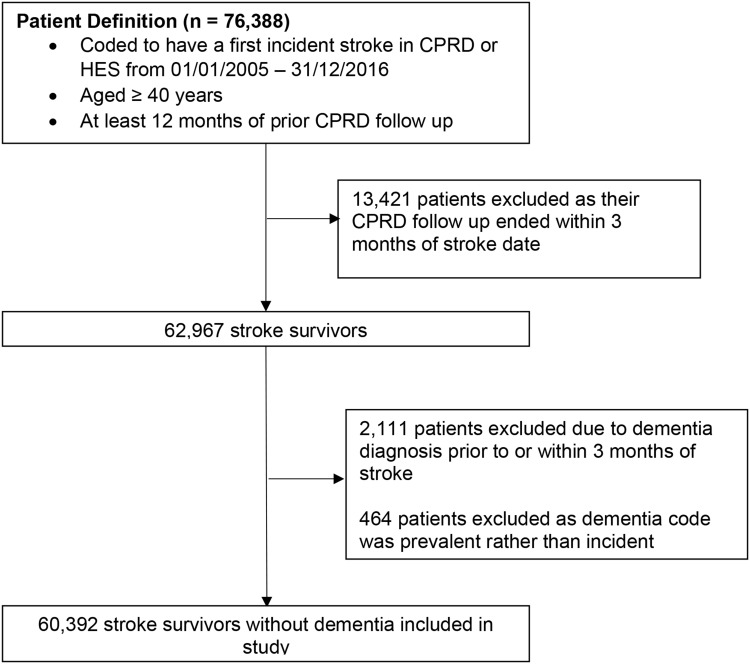
Flowchart of study participants.

We included 60,392 stroke survivors with a median 2.60 years (IQR 0.97–4.75) of follow-up (Figure 1). Just over half were men (51.2%) with a median age of 74.3 years (IQR 63.9–82.4 years). Median consultation frequency was 12.3 per year pre-stroke, rising to 24.2 per year after stroke. A total of 44,057 GP-recorded infections after stroke were identified for 20,969 patients (34.7%), median 2 infections per person (IQR 1–3). First infections comprised 53.6% LRTIs, 33.2% UTIs and 13.1% SSTIs. Linked HES data were available for 64.9%. Table 1 shows study participants’ characteristics overall and by infection status.

**Table 1:** Characteristics of Study Participarticipants

	Total	No Infection	Any GP-Recorded Infection After Stroke*		
			0–3 Months	3 Months–1 Year	1–5 Years
Baseline, N (%)					
All patients	60,392 (100.0)	39,423 (65.3)	3590 (5.9)	6886 (11.4)	10,493 (17.4)
Mean age (SD)	72.6 (12.7)	71.7 (12.9)	76.4 (11.9)	75.4 (12.1)	72.7 (12.1)
Sex					
Male	30,912 (51.2)	21,454 (54.4)	1523 (42.4)	3037 (44.1)	4898 (46.7)
Female	29,480 (48.8)	17,969 (45.6)	2064 (57.6)	3849 (55.9)	5595 (53.3)
Country					
England	45,464 (75.6)	29,892 (75.8)	2700 (75.2)	5290 (76.8)	7772 (74.1)
Wales	5736 (9.5)	3616 (9.2)	324 (9.0)	659 (9.6)	1137 (10.8)
Scotland	7075 (11.7)	4767 (12.1)	401 (11.2)	692 (10.0)	1217 (11.6)
Northern Ireland	1927 (3.2)	1148 (2.9)	165 (4.6)	247 (3.6)	367 (3.5)
Ethnicity					
White	43,526 (72.1)	28,300 (71.8)	2580 (71.9)	5069 (73.6)	7577 (72.2)
South Asian	885 (1.5)	593 (1.5)	50 (1.4)	88 (1.3)	154 (1.5)
Black	543 (0.9)	404 (1.0)	19 (0.5)	47 (0.7)	73 (0.7)
Other/Mixed	520 (0.9)	392 (1.0)	16 (0.5)	46 (0.7)	66 (0.6)
Unknown	14,918 (24.7)	9734 (24.7)	925 (25.7)	1636 (23.8)	2623 (25.0)
Local area deprivation score (quintiles)					
1 (= least deprived)	10,787 (17.9)	6844 (17.4)	708 (19.7)	1277 (18.5)	1958 (18.7)
2	10,638 (17.6)	6935 (17.6)	634 (17.7)	1228 (17.8)	1841 (17.6)
3	12,564 (20.8)	8252 (20.9)	711 (19.8)	1447 (21.0)	2154 (20.5)
4	12,876 (21.3)	8772 (22.3)	711 (19.8)	1293 (18.8)	2100 (20.0)
5 (= most deprived)	13,527 (22.4)	8620 (21.9)	826 (23.0)	1641 (23.8)	2440 (23.3)
Smoking status					
Never	21,273 (36.0)	14,026 (36.6)	1221 (34.2)	2360 (34.9)	3666 (35.1)
Past	25,936 (44.0)	16,396 (42.8)	1729 (48.4)	3156 (46.7)	4694 (45.0)
Current	11,884 (20.0)	7899 (20.6)	623 (17.4)	1249 (18.5)	2073 (19.9)
Alcohol Use (n=54,316)					
None	15,287 (28.1)	9343 (26.7)	1067 (32.4)	2038 (32.8)	2839 (29.1)
Current	39,029 (71.9)	25,708 (73.3)	2231 (67.7)	4176 (67.2)	6914 (70.9)
BMI category					
Underweight	1645 (3.0)	1031 (2.9)	132 (4.0)	213 (3.4)	269 (2.7)
Normal Weight	18,455 (33.9)	12,007 (34.3)	1172 (35.5)	2124 (34.0)	3152 (32.1)
Overweight	20,359 (37.4)	13,297 (38.0)	1180 (35.7)	2231 (35.7)	3651 (37.2)
Obese	13,941 (25.6)	8703 (24.8)	820 (24.8)	1684 (26.9)	2734 (27.9)
Clinical conditions					
History of Diabetes	8978 (14.9)	5431 (13.8)	683 (19.0)	1224 (17.8)	1640 (15.6)
History of Atrial fibrillation	7995 (13.2)	4704 (11.9)	609 (17.0)	1186 (17.2)	1496 (14.3)
History of Myocardial Infarction	4831 (8.0)	2897 (7.4)	366 (10.2)	647 (9.4)	921 (8.8)
History of Depression	3697 (6.1)	2050 (5.2)	348 (9.7)	564 (8.2)	735 (7.0)
Use of medication in 2 years prior to stroke					
Anti-hypertensive	35,781 (59.3)	22,077 (56.0)	2541 (70.8)	4581 (66.5)	6582 (62.7)
Statin	21,837 (36.2)	13,482 (34.2)	1531 (42.7)	2799 (40.7)	4025 (38.4)
Immunosuppressant	6894 (11.4)	3491 (8.9)	726 (20.2)	1308 (19.0)	1369 (13.1)
Post stroke, N (%)					
Antiplatelet use within 90 days	37,148 (61.5)	23,792 (60.4)	2341 (65.2)	4223 (61.3)	6792 (64.7)
New depression post stroke±	5457 (9.6)	2885 (7.7)	383 (11.8)	821 (13.0)	1368 (14.0)

**Notes:** *Defined as any of lower respiratory tract infection, urinary tract infection with antibiotics, or skin/soft tissue infection with antibiotics. Timing is categorised by first infection after stroke. ± N=56,695.

Overall, 4512 (7.5%) patients developed dementia 3 months to 5 years post-stroke, of whom 1406 (31.2%) developed early dementia (rate 35.13 per 1000 PYAR [95% CI 33.34–37.02]) and 3106 (68.8%) developed late dementia (rate 25.80 per 1000 PYAR [95% CI 24.91–26.72]). Both early and late dementia incidence increased with age and was higher in women.

### Infections and Early Post-Stroke Dementia

Early dementia was more common among those with any GP-recorded infection compared to those without (HR 1.44, 95% CI 1.21–1.71). Restricting to those with linked data increased the strength of association (HR 1.53, 95% CI 1.28–1.84). Effect estimates were higher again when hospitalised infections were included either as a primary diagnosis (HR 1.79, 95% CI 1.52–2.11), or as any diagnosis (HR 1.84, 95% CI 1.58–2.14): Table 2, appendix 3. There was no evidence of effect modification with recurrent stroke (p=0.493). Excluding infections in the first 3 months did not affect results (appendix 4)

**Table 2:** Rates of Early and Late Post-Stroke Dementia by Infection Status, and Results from Cox Regression Models

Infection		Total Dementia Cases	Total Person Time	Rate per 1000 Person Years (95% CI)	HR (95% CI)			
					Crude	Sex and Age Adjusted	Fully Adjusted*	P-value **
**Early Dementia**								
All	GP-recorded infections only							
	No Infection	1145	35,284	32.45 (30.63–34.39)	1	1	1	<0.001
	Any Infection	261	4735	55.12 (48.83–62.23)	1.80 (1.57–2.06)	1.44 (1.26–1.65)	1.44 (1.21–1.71)	
Restricted to patients with linked HES	GP recorded infections only							
	No Infection	877	22,657	38.71 (36.23–41.36)	1	1	1	<0.001
	Any Infection	214	3011	71.07 (62.16–81.26)	1.93 (1.66–2.25)	1.57 (1.34–1.82)	1.53 (1.28–1.84)	
	GP recorded and hospital recorded infections							
	No Infection	581	19,052	30.50 (28.11–33.08)	1	1	1	<0.001
	Any Infection	510	6616	77.08 (70.67–84.07)	2.59 (2.30–2.91)	1.99 (1.76–2.24)	1.84 (1.58–2.14)	
	GP recorded and hospital recorded infections where infection was primary diagnosis							
	No Infection	775	21,778	35.59 (33.17–38.18)	1	1	1	<0.001
	Any Infection	316	3891	81.22 (72.74–90.69)	2.40 (2.11–2.74)	1.91 (1.67–2.18)	1.79 (1.52–2.11)	
**Late Dementia**								
All	GP recorded infections only							
	No Infection	2009	84,239	23.85 (22.83–24.92)	1	1	1	0.296
	Any Infection	1097	36,150	30.35 (28.60–32.20)	1.30 (1.20–1.40)	1.07 (0.99–1.15)	1.05 (0.96–1.16)	
Restricted to patients with linked HES	GP recorded infections only							
	No Infection	1562	53,194	29.36 (27.94–30.86)	1	1	1	0.065
	Any Infection	847	22,121	38.29 (35.29–40.96)	1.31 (1.21–1.43)	1.09 (1.00–1.19)	1.10 (0.99–1.22)	
	GP recorded and hospital recorded infections							
	No Infection	955	43,680	21.86 (20.52–23.30)	1	1	1	<0.001
	Any Infection	1454	31,635	45.96 (43.66–48.39)	2.13 (1.96–2.31)	1.66 (1.53–1.80)	1.50 (1.35–1.66)	
	GP recorded and hospital recorded infections where infection was primary diagnosis							
	No Infection	1269	49,488	25.64 (24.27–27.09)	1	1	1	<0.001
	Any Infection	1140	25,827	44.14 (41.65–46.78)	1.75 (1.61–1.90)	1.40 (1.29–1.52)	1.36 (1.23–1.50)	

**Notes:** *Adjusted for age, sex, country, consultation frequency, ethnicity, deprivation, alcohol, smoking, BMI, pre-morbid conditions, pre-stroke use of medication, anti-coagulant prescribing post stroke and depression (pre and post stroke). **Likelihood ratio test.

### Infections and Late Post-Stroke Dementia

For late dementia, while the crude incidence was raised among those who had experienced a GP-recorded infection, adjustment for confounders removed any association (HR 1.05, 95% CI 0.96–1.16). In the sensitivity analyses restricted to those with linked data, there was some evidence that infections were associated with late dementia, with stronger effects seen for exposure definitions that include hospitalised infections (HR 1.36, 95% CI 1.23–1.50 for infection as primary diagnosis) than for GP-recorded infections (HR 1.10, 95% CI 0.99–1.22) (Table 2, appendix 3). Again, there was no evidence of effect modification with recurrent stroke. Excluding infections in the first 3 months did not change results.

### Number and Type of Infections and Post-Stroke Dementia

Increasing number of GP-recorded infections were associated with early dementia (LRT for trend: 0.0002), although only small numbers had ≥2 infections (Table 3). When exposure was expanded to include hospitalised infections, there was an upward trend with increasing number of infections, although confidence intervals overlapped. We saw a strong association between LRTI or UTI as first infection and early dementia (HR 1.46, 95% CI 1.16–1.83, HR 1.61, 95% CI 1.26–2.04 respectively), which was not seen for SSTI, though numbers with dementia were small (n=22). There was an association between an increasing number of GP-recorded infections and late dementia (LRT for trend: 0.023), which was stronger when hospital infections were included (LRT for trend: <0.001). Infection type was not associated with late dementia (p=0.272, appendix 5).

**Table 3:** Rates of Dementia by Number of Infections, and Results from Cox Regression Models

Infection		Total Dementia Cases	Total Person Time	Rate per 1000 Person Years (95% CI)	HR (95% CI)			P-value **
					Crude	Sex and Age Adjusted	Fully Adjusted*	
**Early Dementia**								
All	GP-recorded infections only							
	No Infection	1145	35,284	32.45 (30.63–34.39)	1	1	1	0.0002
	1 infection	211	3851	54.79 (47.88–62.71)	1.77 (1.52–2.05)	1.44 (1.24–1.67)	1.48 (1.23–1.78)	
	2 or more infections	50	884	56.57 (42.87–74.63)	1.94 (1.46–2.58)	1.43 (1.07–1.90)	1.26 (0.88–1.82)	
Restricted to patients with linked HES	GP recorded infections only							
	No Infection	877	22,658	38.71 (36.23–41.36)	1	1	1	<0.001
	1 infection	175	2453	71.35 (61.52–82.74)	1.92 (1.63–2.26)	1.58 (1.34–1.86)	1.59 (1.31–1.92)	
	2 or more infections	39	559	69.83 (51.02–95.58)	1.99 (1.44–2.75)	1.50 (1.09–2.07)	1.31 (0.89–1.94)	
	GP recorded and all hospital infections							
	No Infection	581	19,052	30.50 (28.11–33.08)	1	1	1	<0.001
	1 infection	305	4673	65.28 (58.35–73.03)	2.17 (1.89–2.49)	1.69 (1.47–1.95)	1.57 (1.32–1.87)	
	2 or more infections	205	1944	105.46 (91.97–120.93)	3.65 (3.11–4.28)	2.71 (2.31–3.19)	2.53 (2.06–3.11)	
	GP recorded and primary hospital infections							
	No Infection	775	21,778	35.59 (33.17–39.18)	1	1	1	<0.001
	1 infection	230	3051	75.38 (66.24–85.78)	2.21 (1.90–2.56)	1.78 (1.54–2.07)	1.69 (1.41–2.02)	
	2 or more infections	86	840	102.43 (82.92–126.54)	3.18 (2.54–3.99)	2.38 (1.90–2.99)	2.16 (1.64–2.84)	
**Late Dementia**								
All	GP recorded infections only							
	No Infection	2009	84,239	23.85 (22.83–24.92)	1	1	1	0.023
	1 infection	572	20,900	27.37 (25.22–29.71)	1.16 (1.06–1.28)	1.00 (0.91–1.10)	1.01 (0.90–1.14)	
	2 infections	232	7729	30.02 (26.39–34.14)	1.29 (1.12–1.48)	1.03 (0.90–1.18)	1.03 (0.87–1.21)	
	3 infections	126	3402	37.03 (31.10–44.10)	1.60 (1.34–1.92)	1.23 (1.03–1.47)	1.16 (0.93–1.45)	
	4 infections	61	1668	36.58 (28.46–47.01)	1.59 (1.23–2.06)	1.20 (0.93–1.54)	0.97 (0.69–1.35)	
	5 or more infections	106	2451	43.24 (35.74–52.31)	1.91 (1.56–2.32)	1.41 (1.16–1.72)	1.40 (1.11–1.77)	
Restricted to patients with linked HES	GP recorded infections only							
	No Infection	1562	53,194	29.36 (27.94–30.86)	1	1	1	0.002
	1 infection	428	12,783	33.48 (30.46–36.81)	1.15 (1.03–1.28)	1.00 (0.90–1.12)	1.05 (0.93–1.19)	
	2 infections	187	4762	39.27 (34.03–45.32)	1.35 (1.16–1.58)	1.09 (0.93–1.27)	1.05 (0.88–1.26)	
	3 infections	100	2107	47.46 (39.01–57.74)	1.65 (1.34–2.02)	1.27 (1.03–1.55)	1.25 (0.99–1.57)	
	4 infections	44	1009	43.61 (32.45–58.60)	1.52 (1.12–2.05)	1.14 (0.85–1.55)	1.02 (0.72–1.45)	
	5 or more infections	88	1460	60.28 (48.91–74.28)	2.11 (1.69–2.62)	1.57 (1.26–1.95)	1.55 (1.22–1.97)	
	GP recorded and all hospital infections							
	No Infection	955	43,680	21.86 (20.52–23.30)	1	1	1	<0.001
	1 infection	606	16,527	36.67 (33.86–39.71)	1.70 (1.53–1.88)	1.40 (1.26–1.55)	1.28 (1.13–1.45)	
	2 infections	366	7220	50.69 (45.75–56.16)	2.37 (2.10–2.67)	1.78 (1.58–2.01)	1.70 (1.47–1.96)	
	3 infections	186	3480	53.45 (46.29–61.71)	2.52 (2.15–2.95)	1.86 (1.59–2.18)	1.53 (1.26–1.86)	
	4 infections	110	1774	62.03 (51.45–74.77)	2.94 (2.41–3.59)	2.09 (1.71–2.55)	1.88 (1.48–2.38)	
	5 or more infections	186	2634	70.61 (61.16–81.53)	3.41 (2.90–4.00)	2.44 (2.08–2.86)	2.22 (1.84–2.70)	
	GP recorded and primary hospital infections							
	No Infection	1269	49,488	25.64 (24.27–27.09)	1	1	1	<0.001
	1 infection	539	14,345	37.58 (34.53–40.80)	1.49 (1.34–1.64)	1.25 (1.13–1.38)	1.27 (1.13–1.43)	
	2 infections	262	5664	46.26 (40.98–52.21)	1.84 (1.61–2.11)	1.44 (1.26–1.65)	1.31 (1.11–1.54)	
	3 infections	145	2654	54.63 (46.42–64.29)	2.20 (1.85–2.61)	1.66 (1.39–1.97)	1.50 (1.22–1.85)	
	4 infections	74	1271	58.23 (46.36–73.13)	2.35 (1.86–2.98)	1.71 (1.35–2.17)	1.71 (1.31–2.23)	
	5 or more infections	120	1893	63.39 (53.01–75.81)	2.60 (2.15–3.15)	1.92 (1.58–2.32)	1.86 (1.50–2.32)	

**Notes:** *Adjusted for age, sex, country, consultation frequency, ethnicity, deprivation, alcohol, smoking, BMI, pre-morbid conditions, pre-stroke use of medication, anti-coagulant prescribing post-stroke and depression (pre- and post-stroke). **Likelihood ratio test.

## Discussion

We showed a 44% increase in early dementia among stroke survivors with common infections recorded by the GP after adjusting for other factors, in a UK population cohort of >60,000 individuals. The association was robust to a range of sensitivity analyses and became stronger when hospitalisations with infection were included. While there was little evidence that GP-recorded infection was associated with late dementia, an association was seen when infection hospitalisations were included. These findings suggest that better prevention or management of common infections following stroke may help to improve outcomes including preserving cognitive function, although further work is needed to disentangle the effect of stroke severity on this relationship.

In our study, 7.5% of patients developed dementia from 3 months to five years after stroke, which is at the lower end of the reported range for post-stroke dementia incidence.^2^,^4^ This may be due to its population-based nature. We also identified dementia diagnoses from routine EHRs, in contrast to some other cohort studies which conducted universal cognitive testing. While the positive predictive value of a dementia diagnosis in EHR data is generally high (>75%), sensitivity is lower.^17^ In addition, our study excluded dementia diagnoses in the first 3 months after stroke as acute brain injury post-stroke can temporarily affect cognition.

Estimates of the risk of post-stroke infections vary depending on study population, timing and methods used to identify infections. In a meta-analysis of 87 studies assessing infection in the acute phase after stroke, the pooled infection rate was 30% (95% CI 24% to 36%),^18^ in contrast to our study which found that 6% of patients experienced an infection in the first 3 months. However, the meta-analysis included patients with all premorbid conditions and those who died shortly after stroke, whereas for inclusion in our study, patients had to survive for at least 3 months post-stroke and have no history of dementia. In this population of stroke survivors, we showed that 34.7% had an infection up to 5 years after stroke. Validation studies show that 93% of infections recorded in EHRs can be confirmed by an additional information source.^19^

### Strengths and Limitations of the Study

Using routinely collected electronic health data increases the power and generalisability of findings as well as overcoming some methodological difficulties that may hamper traditional cohort studies of post-stroke dementia, such as ascertainment bias. In addition, there is good capture of demographic and clinical covariates, while completeness and accuracy of diagnoses were enhanced through linkage to hospital data. Diagnoses of stroke, infections and dementia all demonstrate a high positive predictive value in EHRs. Although only around two thirds of dementia patients have a formal diagnosis in their primary care record,^20^ as our study population all had incident strokes and visited the GP regularly, any misclassification of dementia is unlikely to be differential by exposure status. While it is possible that reversible confusion, e.g., induced by infections or persisting beyond three months of stroke could be misclassified as dementia, usual clinical practice is to assess trajectories of cognitive change and functional status over time. In addition, current guidance in England and Wales recommends referral to a specialist dementia diagnostic service such as a memory clinic if dementia is suspected after reversible causes of cognitive change have been investigated,^21^ so this seems less likely. Nevertheless, we cannot exclude some degree of reverse causality, in which individuals with early undiagnosed dementia may develop more infections after stroke, perhaps through poorer nutritional status or self-care, especially as pre-stroke cognition is not routinely captured in EHRs.

In our analysis we were unable to assess severity and location of stroke, which may be predictors of infection and are linked to dementia risk.^3^ We did however limit to those who survived for at least three months which would exclude the most severe strokes. Other studies have shown that both immediate and later infections after stroke are associated with poorer long-term functional outcomes and mortality, independent of any effect of stroke severity.^22^–^24^ In addition, our findings remained similar when excluding infections occurring in the first 3 months, which are likely to be associated with more severe strokes. Only 65% of participants had linked data so contributed to the analyses of hospital infections, but these additional sensitivity analyses confirmed findings for the whole cohort. Therefore, while over diagnosis of infections in primary care due to lack of diagnostic tests could lead to misclassification of exposure, this is unlikely to have significantly affected findings. Some potential confounders such as education are not recorded in routine health data, although we used IMD level as a proxy. Nevertheless, there may be other relevant unmeasured factors such as dysphagia after stroke or urinary catheterization, which could be linked to stroke severity and also to likelihood of infections. We used data from after the introduction of the Quality and Outcomes Framework in 2004, which markedly improved the completeness of primary care recording.^8^

### Mechanisms

Impaired immunity is common after stroke and increases susceptibility to infections.^25^ Systemic inflammation, which impacts the progression of cardiovascular disease, could partly explain the association between infections and post-stroke dementia.^26^,^27^ Major infection can also result in inflammatory brain changes, for example, in multiple sclerosis about one third of all relapses are associated with a systemic infection, with increased immune activation seen on brain imaging.^28^ Infections are also well-recognised to contribute to delirium, which is present in about 20% of all general hospital inpatients, increasing to over 30% in those >80 years old and 27% in stroke patients,^29^,^30^ and is an independent risk factor for dementia.^31^

## Conclusion

Our study found evidence of an association between common infections and post-stroke dementia in a large cohort of UK stroke survivors using linked routine health data. Future research to investigate this relationship would benefit from including markers of stroke severity and repeated standardised measures of cognition to investigate trajectories of a broader range of cognitive outcomes over time. Better understanding of this relationship will help to inform the development and targeting of interventions such as vaccines or early antibiotic use to prevent and treat infections after stroke and perhaps thereby preserve cognitive function.
